# Lactate Metabolism Dysregulation Drives the Pathogenesis of Acute Kidney Injury

**DOI:** 10.3390/metabo16060434

**Published:** 2026-06-22

**Authors:** Yongchen Li, Jingwen Liu, Diman Mai, Renzhi Tan, Chao Wang, Zengnan Mo

**Affiliations:** 1 Center for Genomic and Personalized Medicine, Guangxi Key Laboratory for Genomic and Personalized Medicine, Guangxi Collaborative Innovation Center for Genomic and Personalized Medicine, Guangxi Medical University, Nanning 530021, China; lyc202310112@sr.gxmu.edu.cn; 2 Department of Immunology, School of Basic Medical Sciences, Guangxi Medical University, Nanning 530021, China; 202320036@sr.gxmu.edu.cn (J.L.); 202320032@sr.gxmu.edu.cn (D.M.); 202320883@sr.gxmu.edu.cn (R.T.)

**Keywords:** acute kidney injury, lactate metabolism, lactate dehydrogenase B, metabolic reprogramming, collecting duct cell, single-cell RNA sequencing

## Abstract

Background: Acute kidney injury (AKI) remains a condition with limited effective therapeutic options, partly due to challenges in early diagnosis and timely intervention. While lactate accumulation is a hallmark of ischemic and septic AKI, the underlying mechanisms remain unclear. Methods: This study integrated single-cell RNA sequencing data from AKI patients (GEO database) with lactate metabolism-related genes (LMRGs) to identify key therapeutic targets. Results: Collecting duct (CD) cells exhibited the highest LMRG expression. Machine learning algorithms and validation in bilateral ischemia/reperfusion injury (bIRI) and lipopolysaccharide (LPS)-induced AKI mouse models, as well as hypoxia/reoxygenation (H/R)-stimulated renal cells, identified *Ldhb* as a core gene. Disruption of lactate metabolism via BAY876 (selective GLUT1 inhibitor) or siRNA-mediated *Ldhb* knockdown significantly attenuated kidney injury, reduced inflammatory cytokines (IL-1β, IL-6, TNF-α), and decreased reactive oxygen species in vitro and in vivo. Conclusions: These findings reveal that lactate metabolism is reprogrammed in AKI, particularly in CD cells, and identify LDHB as a novel potential therapeutic target for this condition, though further mechanistic studies are required to establish causality.

## 1. Introduction

Acute kidney injury (AKI) is a clinical syndrome characterized by a rapid decline in renal function over a short period of time, manifesting as oliguria, azotemia, disturbances in water–electrolyte and acid–base balance, and various systemic complications. AKI is highly prevalent among hospitalized patients, with studies reporting an in-hospital occurrence rate of 10–20%, which rises to over 50% in intensive care unit (ICU) patients [1]. In a large-scale multicenter screening study of hospitalized patients, AKI was identified in only 25.8% of affected patients, with an associated in-hospital mortality rate of 12.4% [2]. This high rate of underdiagnosis, combined with substantial mortality, often leads to insufficient monitoring and suboptimal treatment of AKI, thereby increasing risk of progressing to chronic kidney disease (CKD) [3]. The kidneys perform essential physiological functions, including blood filtration, waste excretion, and maintenance of water–electrolyte balance, all of which require considerable energy [4]. When renal injury occurs due to ischemia, sepsis, or nephrotoxic agents, kidney cells, particularly tubular epithelial cells, experience dysregulated energy metabolism, resulting in cell death and transient loss of renal function. Energy metabolism plays a critical role in maintaining cellular homeostasis and enables tubular cells to adapt to external stressors. Metabolic disturbances, particularly a shift from oxidative phosphorylation to glycolysis, are closely associated with the pathogenesis of kidney diseases [5,6,7].

Glucose breakdown is a major energy source for certain renal cell types, particularly those in the medulla such as collecting duct epithelial cells. Under normal physiological conditions, metabolic substrate preferences vary across nephron segments; notably, collecting duct epithelial cells depend more heavily on glucose metabolism to generate ATP [8]. During kidney injury, the central catabolic pathway undergoes alterations that reshape cellular energy acquisition. Studies have indicated that in the early stages of AKI, renal cells shift from physiological oxidative phosphorylation to anaerobic glycolysis, leading to lactic acidosis, mitochondrial dysfunction, and ultimately apoptosis [9]. The renal cortex and medulla have distinct metabolic characteristics. The renal cortex and medulla exhibit distinct metabolic profiles. As a key site of lactate metabolism, the renal medulla undergoes substantial metabolic reprogramming in response to pathological insults, resulting in pathological lactate accumulation and ultimately leading to impaired renal function [10,11]. Modulating glycolysis, and consequently regulating lactate metabolism, has emerged as a therapeutic strategy for various diseases, particularly cardiovascular and neurological disorders. Chen et al. [12] identified a significant correlation between lactate metabolism and serum creatinine levels in patients with diabetic nephropathy. Further evidence suggests that enhanced glycolysis and lactate accumulation may accelerate renal inflammation and fibrosis, whereas targeting the key regulators of lactate metabolism may reduce lactate buildup and mitigate fibrotic progression [13,14]. Given the central role of lactate metabolism in energy homeostasis and its potential as a therapeutic target in kidney injury, investigating its functional implications in AKI is of considerable importance [15].

In recent years, rapid advances in bioinformatics and the widespread adoption of single-cell sequencing technologies have enabled analysis of cellular subpopulations, functions, and communication networks at a single-cell resolution across various diseases, deepening our understanding of pathological mechanisms. In this study, we integrated extensive single-cell transcriptome datasets from the kidneys of patients with AKI to identify reliable lactate metabolism-related target genes. The role of these targets was further validated through in vivo and in vitro experiments, demonstrating that intervention of lactate metabolism significantly alleviates kidney injury.

## 2. Materials and Methods

### 2.1. Data Acquisition and Processing

Single-cell RNA sequencing data from kidney tissues of patients with AKI and controls were obtained from the Gene Expression Omnibus (GEO) database (https://www.ncbi.nlm.nih.gov/geo/info/datasets.html (accessed on 11 December 2025)), specifically from datasets GSE210622 and GSE174220. These datasets collectively included samples from six patients with AKI and six control subjects. Bulk kidney transcriptome data from patients with AKI were sourced from the GEO dataset GSE139061, which contained data from 39 AKI samples and nine control samples. In the GSE210622 dataset, human AKI kidney samples were collected within 1–2 h after death from patients who died of major diseases related to respiratory tract infections; control kidney tissues were obtained from non-AKI patients either postmortem or following nephrectomy. In the GSE174220 dataset, human AKI kidney samples were obtained by renal biopsy, with an appropriate amount of kidney tissue collected. In the GSE139061 dataset, human AKI kidney samples were obtained from renal biopsy, while control kidney samples were derived from nephrectomy. AKI patients in all datasets adhered to the KDIGO diagnostic criteria [16]. A set of 332 lactate metabolism-related genes (LMRGs) was downloaded from the MsigDB database for subsequent analysis.

Single-cell RNA sequencing (scRNA-seq) data were processed using the “Seurat” package (version 5.4.0) in R. Initial quality control involved filtering cells to retain those expressing more than 200 genes and with mitochondrial gene content below 20%, yielding a total of 99,842 high-quality cells for downstream analysis. Subsequent steps included data normalization, scaling, principal component analysis (PCA), and batch effect correction using Harmony (version 1.2.4). Dimensionality reduction and visualization were performed via UMAP, and cell types were annotated using the “SingleR” package (version 2.14.0). To comprehensively evaluate the activity of LMRGs across different cell populations, we identified the cell cluster with the highest overall LMRG activity using five scoring methods: ssGSEA, AUCell, UCell, AddModuleScore, and Singscore. Within this cluster, cells were divided into high- and low-score groups based on the median expression level of LMRGs to identify differentially expressed genes (DEGs) between the two groups. The identified DEGs were subsequently intersected with LMRGs derived from the MsigDB database, thereby identifying a set of core genes designated as LMRG-DEGs that were closely associated with lactate metabolism during AKI progression.

For bulk RNA sequencing data from AKI patient kidneys, differential expression analysis was conducted using the “Limma” package (version 3.64.0). Genes with an adjusted *p*-value < 0.05, and an absolute log_2_ fold change >1 were considered statistically significant and retained for further analysis.

### 2.2. Biological Functions of LMRG-DEGs in AKI Pathogenesis

To investigate pathway-level alterations in the renal transcriptome associated with AKI, Gene Set Enrichment Analysis (GSEA) was performed based on DEGs identified from the GSE139061 dataset (AKI vs. control).

Based on the thresholds of *p* < 0.05, FDR < 0.05, and |NES| > 1, we identified and focused on the top three significantly enriched pathways that were most strongly linked to LMRG-DEG expression. In parallel, KEGG enrichment analysis was conducted to elucidate the biological functions of the DEGs, and the top five significantly enriched pathways were selected for visualization.

Furthermore, single-sample gene set enrichment analysis (ssGSEA) was applied to the GSE139061 dataset using the CIBERSORT algorithm to characterize alterations in the immune microenvironment of AKI kidneys and explore the potential regulatory effects of the identified hub genes on immune cell composition.

### 2.3. Machine Learning Algorithms

To further identify potential lactate metabolism-related target genes in AKI, the intersecting genes between CD-DEGs and LMRGs, combined with the expression matrix of differential genes from the GSE139061 dataset, were analyzed using two machine learning algorithms: Least Absolute Shrinkage and Selection Operator (LASSO) and Support Vector Machine-Recursive Feature Elimination (SVM-RE). Overlapping genes identified by both algorithms were examined for their functional and biological significance in AKI.

### 2.4. Animal Experiments

Eight-week-old male C57BL/6 mice (8 weeks old) were purchased from SPF (Beijing) Biotechnology Co., Ltd. (Beijing, China; License No.: 1103242). All animals were maintained under specific pathogen-free (SPF) conditions with a 12/12 h light/dark cycle and ad libitum access to food and water. A total of 36 mice were used in this study. Mice were randomly assigned to each group (n = 6 per group). All animal procedures were approved by the Ethics Committee of Guangxi Medical University (Approval No. 202505001). All methods were reported in compliance with the ARRIVE guidelines.

### 2.5. Animal Models of Acute Kidney Injury

Mice were not fasted before ischemia–reperfusion surgery. The IRI surgery was performed as described previously [17]. Briefly, under gaseous isoflurane anesthesia, an incision was made on the back to expose both kidneys, and the renal arteries and veins were clamped for 30 min. The mice were maintained at a constant body temperature of 37 °C throughout the procedure. In the sham-operated group, only anesthesia and muscle incisions were performed. The animals were divided into three groups (n = 6 per group) as follows: control, IRI, and IRI + BAY 876. For the LPS-induced AKI model, mice received an intraperitoneal injection of LPS (10 mg/kg) to induce acute kidney injury. LPS from Escherichia coli O111:B4 (Lipopolysaccharide, Ambeed, Shanghai, China, A1228298). The control mice received an equal volume of sterile normal saline as the vehicle control. The animals were divided into three groups (n = 6 per group) as follows: control, LPS, and LPS + BAY 876. BAY876 (BAY-876, MedChemExpress, Monmouth Junction, NJ, USA, HY-100017), a selective GLUT1 inhibitor, was administered via oral gavage (5 mg/kg) 24 h before surgery. All surgeries were performed under strict sterile conditions in the SPF animal facility, and no antibiotics were administered postoperatively. All mice were euthanized 24 h after surgery. Blood samples were collected via orbital sinus puncture, and kidney tissues were harvested for further analysis.

### 2.6. Histological Analysis

Kidney tissues were fixed in paraformaldehyde, dehydrated, and embedded in paraffin. Sections were stained with hematoxylin and eosin (H&E). Tubular injury was assessed in a blinded manner by two renal pathologists, who evaluated 20 randomly selected fields per section. The degree of tubular damage was graded on a scale of 0 to 4 as follows: 0, normal kidney; 1, ≤5% tubular necrosis; 2, 5–25% tubular necrosis; 3, 26–75% tubular necrosis; 4, >75% tubular necrosis.

### 2.7. Cell Culture

293T cells and HK2 cells were obtained from Wuhan Pricella Biotechnology Co., Ltd. (Wuhan, China). 293T cells were cultured in DMEM medium (Wisent, Saint-Jean-Baptiste, QC, Canada) supplemented with 10% fetal bovine serum (Wisent) and 1% penicillin–streptomycin (Wisent). HK2 cells were maintained in MEM medium (Gibco, Grand Island, NY, USA) containing 10% fetal bovine serum and 1% penicillin–streptomycin. Both cell lines were cultured in a humidified incubator with 5% CO_2_ at 37 °C (Thermo Fisher Scientific, Waltham, MA, USA).

### 2.8. siRNA Transfection

GenePharma designed the interference RNA sequence, including four interference sequences: si-524, si-1003, si-428, and si-811. After the transfection system is developed and the cell density in the 6-well plate reaches 30% to 60%, transfection can begin. According to the instructions, 100 pmol of siRNA, 125 mL of Buffer, and 7.5 mL of siRNA-mate plus (GenePharma, Suzhou, China) were added. The mixture was thoroughly mixed. A 6-well plate containing HK-2 cells was dropped with the transfection agent, shook gently, and then cultivated in a cell incubator for 24 h for RT-qPCR detection of transfection efficiency. We found that the si-428 sequence had the best knockdown efficiency and ultimately selected it for subsequent experiments. The qPCR validation results of the siRNA are shown in Supplementary Table S1H.

### 2.9. Hypoxia/Reoxygenation (H/R) Model

293T and HK2 cells were cultured to 80–90% confluence, after which the medium was replaced with D-glucose-free medium. Cells were incubated under hypoxic conditions (1% O_2_) for 6 h, then switched back to normal medium and cultured under normoxic conditions for 3 h to achieve reoxygenation.

### 2.10. Immunofluorescence Staining

Paraffin-embedded tissue sections were taken and baked at 60 °C for about 1 h. After deparaffinization with xylene, they were hydrated through a graded ethanol series to distilled water. Heat-induced antigen retrieval was performed, followed by cooling to room temperature and rinsing with PBS. Permeabilization was carried out with 0.1–0.3% Triton X-100 for 10–15 min as needed. Blocking was done with 5% BSA or 10% normal serum at room temperature for 30–60 min. Primary antibody (diluted according to the instructions) was added, and sections were incubated overnight at 4 °C in a humid box. After washing with PBS, the corresponding fluorescently labeled secondary antibody was added and incubated at room temperature in the dark for about 1 h. Sections were washed with PBS, counterstained with DAPI for about 5–10 min, washed again with PBS, and mounted with anti-fluorescence quenching mounting medium. Images were observed and captured using a fluorescence microscope. A negative control without the primary antibody was set up to assess non-specific background, and all operations were performed in the dark as much as possible. The anti-NGAL antibody (ab216462) and anti-TIM-1 (also known as KIM-1) antibody (ab316854) were both purchased from Abcam (Cambridge, UK).

### 2.11. Quantitative Real-Time PCR (qRT-PCR)

The siRNA-transfected cells were harvested 48 h after transfection and the total RNA was extracted using FastPure Cell/Tissu Total RNA Isolation Kit according to the manufacturer’s protocol (Vazyme, Nanjing, China). Total RNA (1000 ng) was reverse transcribed into cDNA using PrimeScript RT Master Mix (TaKaRa, Shiga, Japan). Then, qRT-PCR was performed using FastStart Essential DNA Green Master for qPCR in Quant Studio 6 Pro according to the manufacturer’s instructions (Roche, Basel, Switzerland). The specific qRT-PCR cycling conditions were 95 °C for 10 min, followed by 45 cycles of 95 °C for 10 s, 60 °C for 10 s and 72 °C for 10 s. The relative mRNA expression levels of the target genes were calculated by the 2−ΔΔCt method using glyceraldehyde-3-phosphate dehydrogenase (GAPDH) as an internal control. The same operation is performed after grinding the kidney tissue. The list of primers used in the qPCR experiments is provided in Supplementary Table S1I.

### 2.12. ROS and Elisa Assay

Mouse blood samples were collected via orbital sinus or cardiac puncture and centrifuged at 3500 rpm for 20 min at ambient temperature to obtain serum. Serum levels of blood urea nitrogen, creatinine, and lactate were measured using commercial reagent kits (Nanjing Jiancheng Bioengineering Institute, Nanjing, China) according to the manufacturer’s protocols.

ROS production was determined using a ROS assay kit (AIDISHENG, Yancheng, China) following the manufacturer’s instructions. Briefly, HK2 and HEK 293T cells were cultured in 6-well plates for 24 h, then treated with BAY-876 at the indicated concentrations for 24 h, followed by stimulation with 10 μg/mL LPS for an additional 12 h. Subsequently, the cells were washed with PBS (biosharp, Hefei, China) and the highly sensitive 2′,7′-dichlorodihydrofluorescein diacetate (DCFH-DA) dye working solution was added. The cells were incubated at 37 °C for 30 min. As a positive control for ROS, the cells were treated with H_2_O_2_.

All in vitro experiments were independently repeated at least three times as biological replicates, and in vivo experiments included six biological replicates per experimental group to ensure the robustness of the results.

### 2.13. Statistical Analysis

Statistical analysis was performed using GraphPad Prism 10.0 and R software (version 4.2.3). Normality of continuous variables was assessed using the Shapiro–Wilk test. Normally distributed data were presented as mean ± standard deviation (SD), and comparisons between two independent groups were performed using the unpaired two-sample t-test. Non-normally distributed data were expressed as median (interquartile range, IQR), and group comparisons were conducted using the Wilcoxon rank-sum test. For comparisons involving more than two groups, one-way analysis of variance (ANOVA) followed by Tukey’s post hoc test was used for normally distributed data, while the Kruskal–Wallis test followed by Dunn’s post hoc test was applied for non-normally distributed data. Statistical significance was set at *p* < 0.05.

## 3. Results

### 3.1. Data Processing and Analysis

**Figure 1:**
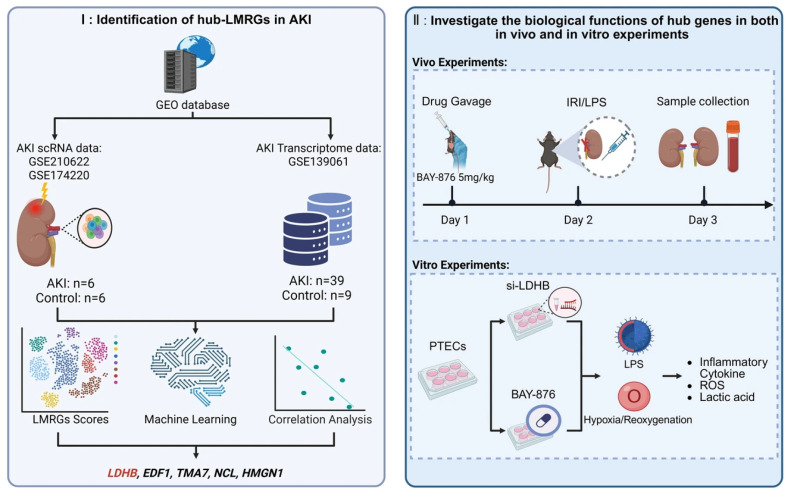
Schematic diagram illustrating the identification and functional investigation of hub-LMRGs in acute kidney injury (AKI).

**Figure 2:**
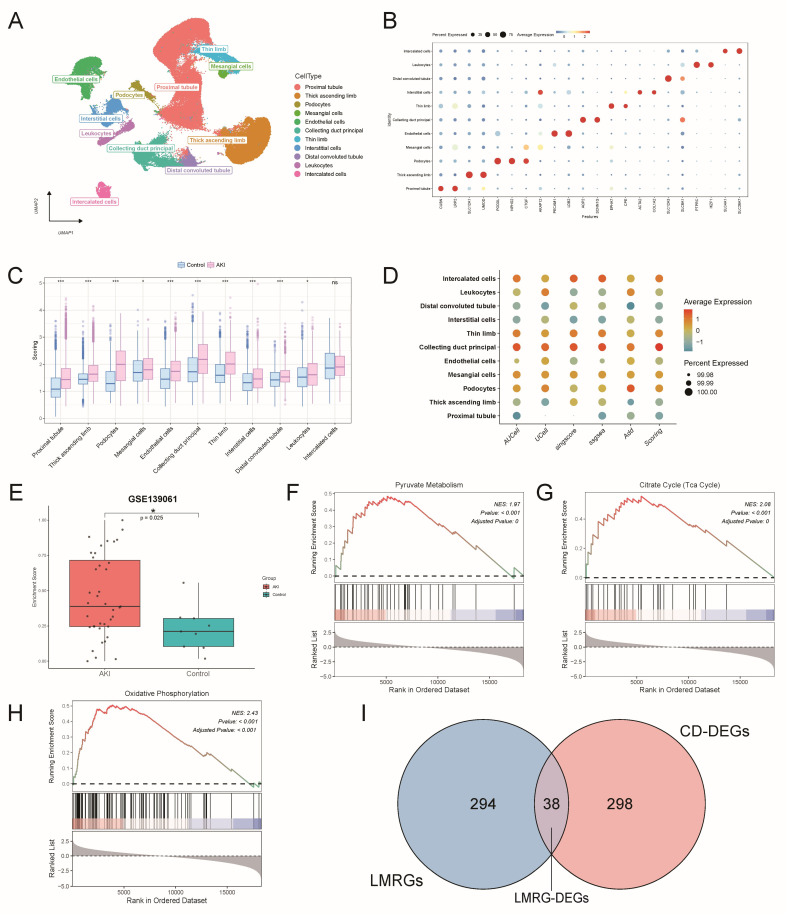
Lactate metabolism is reprogrammed in AKI, with collecting duct cells identified as the primary site. (**A**) UMAP visualization of 11 annotated cell clusters from human AKI kidney samples. (**B**) Dot plot showing the expression of canonical marker genes used for cell type annotation. (**C**) Box plot comparing the expression scores of LMRGs in each cell cluster between AKI and control samples. (**D**) Comprehensive LMRGs activity scores across cell types calculated by five algorithms. (**E**) Elevated LMRGs expression in bulk AKI transcriptomes. (**F**–**H**) Gene Set Enrichment Analysis (GSEA) of AKI transcriptomic data. Enrichment plots show significant upregulation in AKI of key lactate metabolism-related pathways, including (**F**) Pyruvate Metabolism, (**G**) Citrate Cycle (TCA Cycle), and (**H**) Oxidative Phosphorylation (NES and *p*-value indicated for each). (**I**) Identification of lactate metabolism-related differentially expressed genes (LMRG-DEGs) in collecting duct cells. *, *p* < 0.05; ***, *p* < 0.001.

The workflow of this study is illustrated in Figure 1. Initially, single-cell data from AKI kidney tissues underwent standardization, normalization, and batch-effect removal, and the results are shown in Figure S1A–C. Following dimensionality reduction, 14 cell clusters were identified (Figure S1D). Annotation based on marker genes yielded 11 distinct cell types (Figure 2A,B, Supplementary Table S1A). Subsequently, the average expression levels of the 332 lactate metabolism-related genes [18] (LMRGs) across the cell clusters were visualized using box plots (Figure 2C). We observed that major cell populations injured during AKI—including proximal tubular (PT) cells, collecting duct (CD) cells, T lymphocytes (TL), thick ascending limb (TAL) cells, and endothelial cells (ECs)—exhibited significant alterations in lactate metabolism. We then comprehensively assessed the LMRGs activity scores across cell types using five methods: ssGSEA, AUCell, UCell, AddModuleScore, and Singscore. CD cells consistently displayed high lactate metabolism scores across the multiple scoring approaches (Figure 2D, Supplementary Table S1B). Similarly, analysis of bulk transcriptomic data from AKI kidneys revealed significantly elevated expression of LMRGs in the AKI group compared to controls (Figure 2E). Gene Set Enrichment Analysis (GSEA) of differentially expressed genes (DEGs) from the transcriptomic dataset indicated that pathways closely associated with lactate metabolism, such as oxidative phosphorylation, pyruvate metabolism and TCA cycle, were significantly upregulated in the AKI group (Figure 2F–H).

In summary, integrative analysis of multi-omics data from AKI kidney samples consistently demonstrated enhanced lactate metabolism during AKI, predominantly localized in CD cells, which aligns with findings from previous studies by Xu et al. [19]. Xu et al. found that, compared with proximal tubular cells, CD cells are known to rely more on glycolysis than on oxidative phosphorylation, and that CD cells undergo significant metabolic alterations during renal ischemia. Finally, we intersected CD differentially expressed genes (CD-DEGs) with LMRGs and identified 38 key lactate metabolism-related differentially expressed genes (LMRG-DEGs) involved in AKI progression (Figure 2I, Supplementary Table S1C).

### 3.2. Screening for Core Genes Associated with Lactate Metabolism in AKI

**Figure 3:**
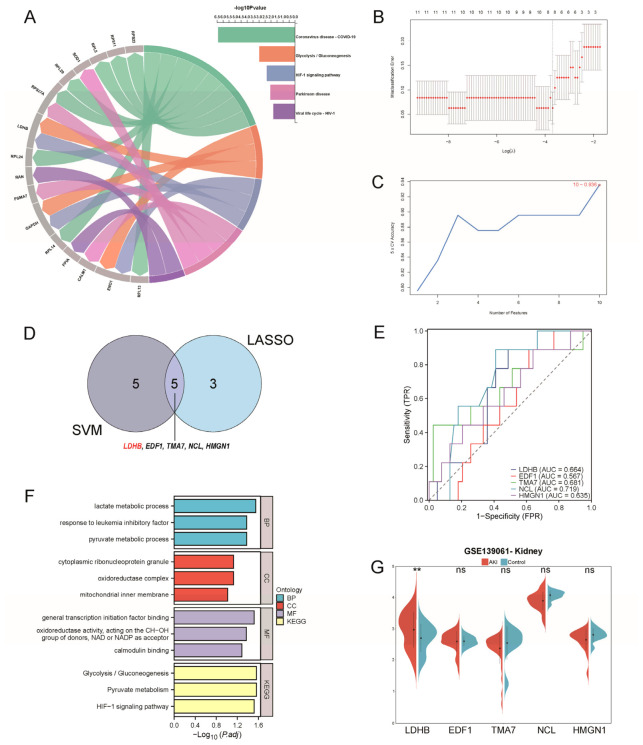
Identification and validation of five hub-LMRG-DEGs in AKI. (**A**) KEGG pathway enrichment analysis of the 38 LMRG-DEGs. Top five significantly enriched pathways and their associated genes are displayed. (**B**) LASSO (Least Absolute Shrinkage and Selection Operator) coefficient profiles of the 38 LMRG-DEGs. (**C**) SVM-RFE (Support Vector Machine-Recursive Feature Elimination) analysis showing the minimal feature set with optimal classification accuracy. (**D**) Identification of five hub-LMRG-DEGs by intersecting genes selected from LASSO and SVM-RFE results. (**E**) Diagnostic performance of hub-LMRG-DEGs for AKI assessed by ROC curves. (**F**) GO and KEGG enrichment analysis of the five hub-LMRG-DEGs reveals their associated biological functions. (**G**) Differential expression of hub-LMRG-DEGs in the AKI transcriptomic dataset. **, *p* < 0.01; ns, not significant.

Following the identification of the 38 intersecting genes, Kyoto Encyclopedia of Genes and Genomes (KEGG) pathway enrichment analysis was performed. The top five enriched pathways along with their associated genes are presented in Figure 3A. To further identify the core genes, the 38 genes were analyzed using two machine learning algorithms: Least Absolute Shrinkage and Selection Operator (LASSO) and Support Vector Machine (SVM) (Figure 3B,C, Supplementary Table S1D,E). Integration of the results from both methods led to the identification of five core genes implicated in lactate metabolism during AKI: *LDHB*, *EDF1*, *TMA7*, *NCL*, and *HMGN1* (Figure 3D). Their potential as diagnostic indicators was evaluated by constructing receiver operating characteristic (ROC) curves based on the expression matrix from the GSE139061 dataset. The results suggested that all five genes exhibited good discriminatory ability in distinguishing AKI samples from controls within this dataset, although external validation in independent cohorts is needed to confirm their diagnostic utility (Figure 3E). Subsequent KEGG and Gene Ontology (GO) enrichment analyses of these five core genes revealed that they are significantly associated with biological processes and pathways related to lactate metabolism, glycolysis, and energy metabolism (Figure 3F). Finally, expression levels were validated using a bulk kidney transcriptome dataset from patients with AKI. Among them, LDHB showed a significant increase in expression in AKI samples compared to that in the controls (Figure 3G).

### 3.3. Investigating the Biological Functions of Core LMRGs

**Figure 4:**
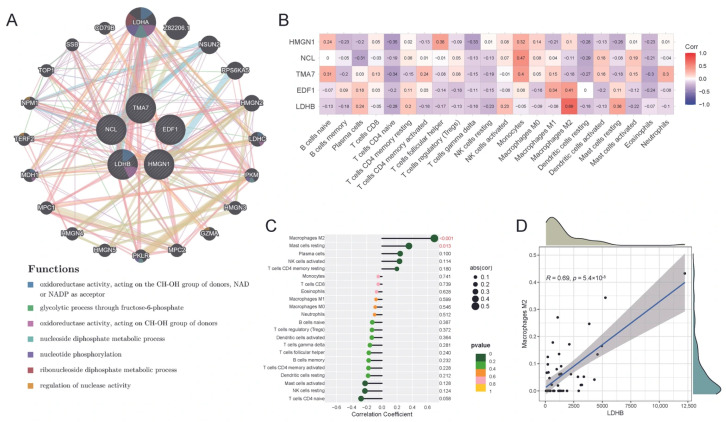
Biological functions and immune correlation of hub LMRGs in AKI. (**A**) Protein–protein interaction network of the five hub-LMRGs constructed using the GENEMANIA database. (**B**) Single-sample gene set enrichment analysis (ssGSEA) showing the correlation between the five hub-LMRGs and the infiltration levels of various immune cell subtypes in AKI. (**C**,**D**) Lollipop and scatter plots demonstrate a significant positive correlation between *LDHB* expression and M2 macrophage infiltration.

The protein functions of the five core LMRGs were explored using the GENEMANIA database. Analysis revealed that *LDHB* plays a significant role in various energy metabolism processes, particularly through its interaction with *LDHA*, which regulates oxidoreductase activity and participates in NAD+-related energy metabolism pathways (Figure 4A). To investigate the potential immunometabolic regulatory roles of these five core LMRGs in AKI, we performed ssGSEA to assess their associations with different immune cell subtypes (Figure 4B). Among the findings, the *LDHB* gene showed a significant and positive correlation with M2 macrophages (Figure 4C,D, Supplementary Table S1F,G). Given that M2 macrophages are generally associated with anti-inflammatory responses and tissue repair, this correlation raises the possibility that *LDHB* may be linked to immunomodulatory processes in AKI; however, further functional studies are required to determine whether and how *LDHB* directly influences macrophage polarization or function.

### 3.4. Inhibition of the Lactate Metabolic Pathway Alleviates Kidney Injury and Systemic Inflammation

**Figure 5:**
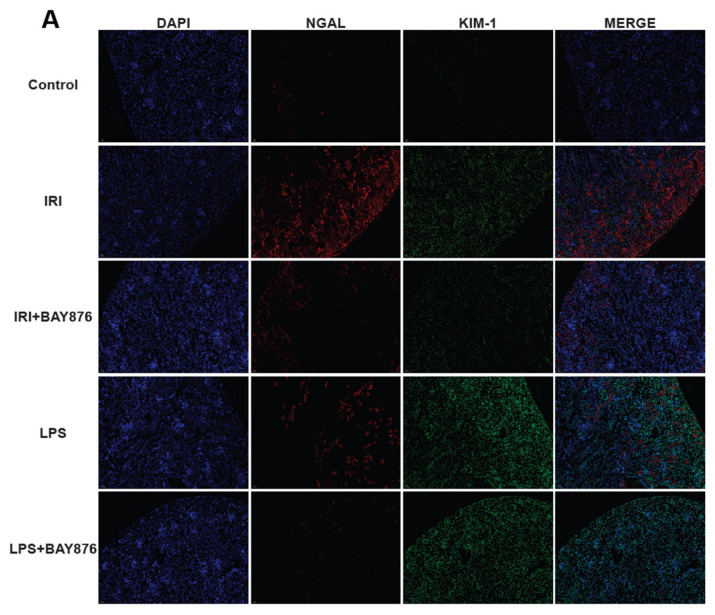

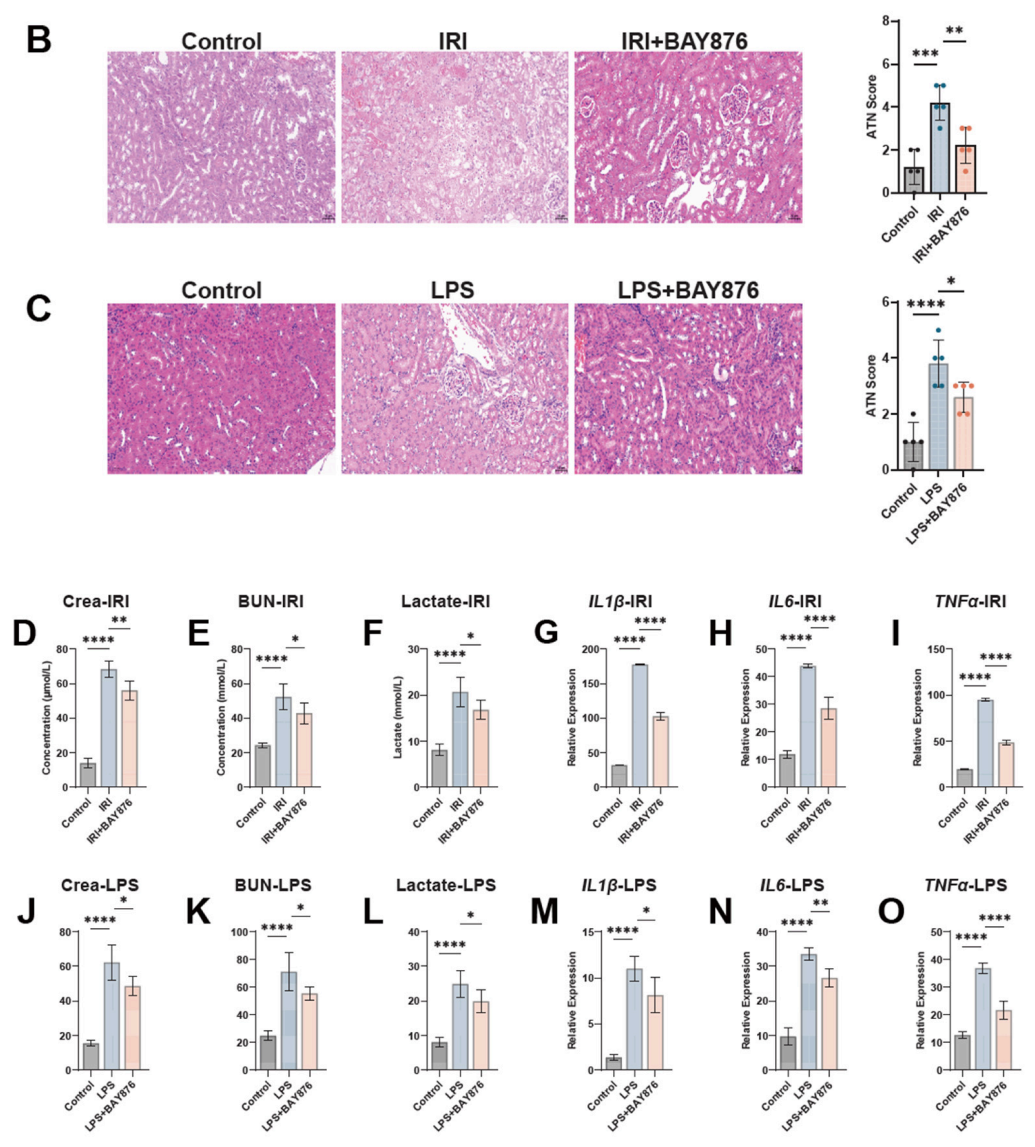
BAY876 alleviates IRI- and LPS-induced acute kidney injury by inhibiting lactate metabolism. (**A**) Immunofluorescence staining of kidney injury markers (KIM-1, NGAL) in renal tissues from each group (Control, IRI, IRI+BAY876, LPS, LPS+BAY876). (**B**,**C**) Representative H&E-stained kidney sections and corresponding acute tubular necrosis (ATN) scores in the IRI (**B**) and LPS (**C**) model. (**D**–**F**) Serum creatinine (**D**), blood urea nitrogen (BUN) (**E**), and lactate (**F**) levels in the IRI model. (**G**–**I**) Serum levels of inflammatory cytokines IL-1β (**G**), IL-6 (**H**), and TNF-α (**I**) in the IRI model. (**J**–**L**) Serum creatinine (**J**), BUN (**K**), and lactate (**L**) levels in the LPS model. (**M**–**O**) Serum levels of inflammatory cytokines IL-1β (**M**), IL-6 (**N**), and TNF-α (**O**) in the LPS model. *, *p* < 0.05; **, *p* < 0.01; ***, *p* < 0.001, ****, *p* < 0.0001.

To investigate the role of the lactate metabolic pathway in AKI, we used BAY876 [20], a selective inhibitor of glucose transporter 1 (GLUT1), administered 24 h prior to injury induction to evaluate its protective effects against the development of AKI in ischemia–reperfusion injury (IRI) and LPS-induced AKI models. In the IRI model, BAY876 treatment significantly reduced the expression of the kidney injury markers KIM-1 [21] and NGAL [22] (Figure 5A), mitigated the degree of tubular damage, and markedly lowered the acute tubular necrosis (ATN) score (Figure 5B). Concurrently, BAY876 significantly decreased serum levels of creatinine, blood urea nitrogen (BUN), and lactate (Figure 5D–F), and suppressed the release of inflammatory cytokines IL-1β, IL-6, and TNF-α (Figure 5G–I). Similarly, BAY876 exerted a protective effect in an LPS-induced AKI model. It also reduced the expression of renal injury markers (Figure 5A), alleviated histopathological damage (Figure 5C), and lowered the ATN scores (Figure 5C). Furthermore, serum creatinine, BUN, and lactate levels in the BAY876-treated group were significantly lower than those in the LPS group (Figure 5J–L). The expression of inflammatory cytokines IL-1β, IL-6, and TNF-α was also significantly suppressed (Figure 5M–O).

In summary, BAY876 significantly attenuated the severity of IRI- and LPS-induced AKI and reduced the systemic inflammatory response by inhibiting the lactate metabolism pathway.

### 3.5. Inhibition of Lactate Uptake or Synthesis Pathways Effectively Alleviates Injury Phenotypes in Renal Tubular Epithelial Cells

**Figure 6:**
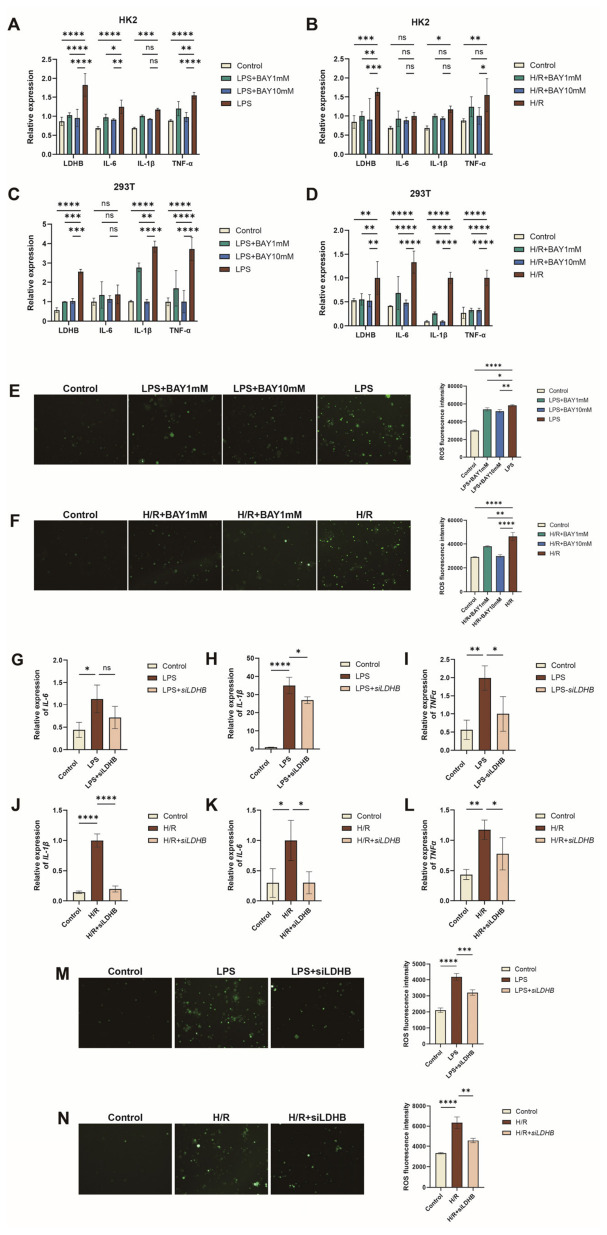
Inhibition of lactate metabolism mitigates inflammatory response and oxidative stress in renal tubular epithelial cells. (**A**,**B**) mRNA expression levels of LDHB, IL-6, IL-1β, and TNF-α in HK2 cells treated with increasing concentrations of BAY876 under LPS stimulation (**A**) or hypoxia/reoxygenation (H/R) (**B**). (**C**,**D**) mRNA expression levels of LDHB, IL-6, IL-1β, and TNF-α in 293T cells treated with BAY876 under LPS (**C**) or H/R (**D**) conditions. (**E**,**F**) Levels of reactive oxygen species (ROS) in 293T cells treated with BAY876 under LPS (**E**) or H/R (**F**) stimulation. (**G**–**L**) mRNA expression levels of IL-6 (**G**,**J**), IL-1β (**H**,**K**), and TNF-α (**I**,**L**) in HK2 cells transfected with siLDHB or scramble control, followed by LPS (**G**–**I**) or H/R (**J**–**L**) stimulation. (**M**,**N**) ROS levels in HK2 cells transfected with siLDHB under LPS (**M**) or H/R (**N**) stimulation. *, *p* < 0.05; **, *p* < 0.01; ***, *p* < 0.001, ****, *p* < 0.0001.

In vitro, H/R and LPS were used as interventions to model renal cell injury. In HK2 cells, treatment with the lactate transporter inhibitor BAY876 reduced mRNA expression of *LDHB*, *IL-6*, and *TNF-α* induced by LPS or H/R in a dose-dependent manner. The expression of IL-1β also exhibited a dose-dependent decreasing trend, although statistical significance was not reached at all concentrations (Figure 6A,B). A similar pattern was observed in 293T cells, wherein BAY876 significantly suppressed the expression of *LDHB* and pro-inflammatory cytokines (Figure 6C,D). Furthermore, BAY876 treatment significantly lowered the levels of reactive oxygen species (ROS) produced by 293T cells under both injury conditions (Figure 6E,F), providing direct evidence that targeting lactate transport alleviates oxidative stress. Our experimental results demonstrated that BAY876 treatment significantly reduced *LDHB* mRNA expression in a dose-dependent manner under both LPS and H/R conditions, suggesting that upstream inhibition of glucose uptake may influence the expression of this key lactate-metabolizing enzyme.

To further elucidate the role of lactate production, we knocked down the key lactate synthetic enzyme *LDHB* in HK2 cells. *LDHB* knockdown significantly inhibited the upregulation of IL-1β and *TNF-α* gene expression triggered by LPS or H/R stimulation, while *IL-6* expression also showed a consistent decreasing trend (Figure 6G–L). Additionally, *LDHB* knockdown markedly reduced ROS accumulation in both injury models (Figure 6M,N).

In summary, these in vitro findings demonstrate that interventions targeting lactate metabolism, either by inhibiting its cellular uptake (via BAY876) or reducing its synthesis (via siLDHB), effectively mitigated the inflammatory response and oxidative stress in renal tubular epithelial cells. These results provide direct cellular evidence supporting the protective role of lactate-related pathway modulation. One limitation is that 293T cells are derived from embryonic kidney, but the consistent results from HK2 cells support our main conclusions. Additionally, cell viability data and deeper metabolic parameters (e.g., NAD^+^/NADH ratio, LDH enzyme activity) were not assessed in this study, representing a limitation of the current work. Future studies using in vivo models or primary renal tubular cells would be valuable to further validate these findings. We did not assess cell viability or measure antioxidant enzyme activities in our in vitro experiments; thus, our ROS data reflect a balance between production and scavenging rather than direct evidence of cytoprotection. Future studies incorporating cell viability and antioxidant capacity assays are needed to validate the protective mechanisms of BAY876 and siLDHB.

## 4. Discussion

Acute kidney injury is prevalent worldwide and a leading cause of in-hospital mortality. Early prediction of AKI-related clinical events and timely intervention in high-risk patients can significantly improve prognosis [23]. Given the high prevalence and mortality of in-hospital AKI, early identification and treatment are crucial for successful outcomes [24,25]. In this study, by analyzing large-scale single-cell RNA sequencing data from AKI patients, we identified CD cells as a principal cell population exhibiting high expression of LMRGs during AKI. Using machine learning approaches, we further identified *LDHB* as a core LMRG in CD cells. Using IRI- and LPS-induced mouse AKI models, we investigated the expression of hub LMRGs and evaluated the therapeutic potential of interfering with the lactate metabolic pathway. Finally, using in vitro models that simulate key pathological features of AKI, namely hypoxia/reoxygenation (H/R) and LPS-induced inflammatory stress, we demonstrated that *LDHB* knockdown significantly alleviated cellular damage.

Lactate, once regarded merely as a byproduct of glycolysis, is increasingly being recognized as an important signaling molecule. Lactate can act as a signaling molecule by binding to the G-protein-coupled receptor GPR81, which is expressed in various tissues including adipose tissue and immune cells. Upon lactate binding, GPR81 suppresses cAMP production and downstream signaling, thereby inhibiting lipolysis and modulating inflammatory responses [26,27]. Studies have shown that lactate produced in tissues, such as skeletal muscle, can be transported via the bloodstream to organs rich in *LDHB* and mitochondria, including the heart, kidneys, and brain, where it is oxidized and utilized [28]. This process plays a key role in post-exercise recovery and the interorgan coordination of energy substrates [29,30]. As a signaling molecule, lactate exerts diverse biological functions, including regulation of energy metabolism [31,32], modulation of cardiovascular function [33,34], influence on gene expression [35], and alteration of cognitive performance [36]. In the kidney, cytokine storms and tissue ischemia–hypoxia are critical factors in acute injury. Lactate is involved in the process of inflammatory injury by activating multiple cellular signaling pathways and modulating inflammatory progression. Tissue ischemia disrupts the supply of oxygen and glucose, resulting in lactate accumulation and subsequent metabolic dysregulation such as disturbances in glucose metabolism [37]. Research has shown that under conditions of hypoperfusion in cardiac and cerebral tissues, lactate can induce the release of classic inflammatory cytokines such as TNF-α and IL-6, thereby amplifying inflammation [38,39]. Under stressful conditions, tissue hypoxia, altered glycolytic pathways, and lactate accumulation create a vicious cycle that exacerbates inflammation and damages multiple organs, including the kidneys. Nevertheless, this study offers new therapeutic insights. Targeting lactate metabolism may represent a promising strategy for mitigating renal inflammation and AKI. Our findings provide further evidence supporting the potential of modulating lactate-related pathways as a novel intervention for AKI management.

BAY-876 is a highly selective, orally bioavailable inhibitor of GLUT1. Its primary pharmacological action is to suppress GLUT-1-mediated glucose uptake, thereby interfering with cellular energy metabolism—particularly by inhibiting aerobic glycolysis under pathological conditions [40]. In immune cells, BAY-876 significantly reduces glucose uptake in activated T cells and macrophages (by 41% and 15%, respectively), decreases glycolytic proton efflux rate (glycoPER) and lactate production, and reprograms cellular metabolism from glycolysis dependence toward greater reliance on oxidative phosphorylation (OXPHOS) to maintain ATP homeostasis [41]. This metabolic shift not only preserves the basal energy supply but also attenuates pro-inflammatory phenotypes in various immune cells. Studies in renal LPS-induced AKI models have confirmed that BAY-876 significantly alleviates kidney tissue damage, suppresses inflammatory cytokine infiltration by immune cells, and improves renal function. Therefore, the protective effects observed with BAY-876 in our IRI and LPS models demonstrate that intervention at the level of glycolysis and upstream lactate synthesis confers renoprotection. Furthermore, siRNA-mediated LDHB knockdown in renal cells attenuated H/R- and LPS-induced inflammation and oxidative stress, confirming LDHB itself as a functional AKI target and key lactate pathway node driving AKI pathogenesis, rather than effects solely from upstream GLUT1 inhibition. Collectively, previous studies and the present investigation demonstrate that BAY-876 exerts a protective effect on the kidney across a variety of injury models, offering a novel therapeutic strategy for the treatment of renal injury. However, systemic GLUT1 inhibition with BAY876 may involve extra-renal contributions, and future studies using kidney-specific conditional knockout of GLUT1 or LDHB are needed to confirm renal-autonomous effects.

LDH is an important oxidoreductase whose core function is to catalyze the interconversion between lactate and pyruvate. The two most critical subunits of LDH are the M (encoded by *LDHA*) and H subunits (encoded by *LDHB*). Both LDHA and LDHB catalyze the reversible conversion between pyruvate and lactate, with LDHA favoring pyruvate reduction and LDHB favoring lactate oxidation under physiological conditions. [42]. *LDHA* and *LDHB* exhibit distinct expression patterns in the kidney. As shown in a study by Azushima et al. [43], *LDHA* is moderately expressed in normal kidneys, with the most prominent expression observed in the proximal tubules and glomerular endothelial cells. In contrast, *LDHB* is most strongly expressed in the distal nephron, including the distal convoluted tubule, loop of Henle, and principal cells, and is abundantly present in glomerular parietal epithelial cells. Intriguingly, single-cell sequencing analysis of diabetic nephropathy mouse kidneys showed that *LDHB* was upregulated in CD cells, a finding consistent with the results of the present study. This suggests pronounced glycolytic alterations in CD cells and indicates that *LDHA* and *LDHB* respond differently to injury [43]. Furthermore, a single-cell analysis comparing healthy and IRI mouse kidneys revealed that *LDHB* expression increases following renal injury, exhibiting a pattern of rapid decline followed by a gradual recovery over time [44]. Similarly, in this study, *LDHB* expression showed a significant positive correlation with M2 macrophages, suggesting that *LDHB* possesses a potential immunoregulatory mechanism. As an effective interventional target, *LDHB* has already been applied in the treatment of various diseases, although most studies focus on malignancies. For example, targeting *LDHB* has been shown to significantly inhibit the progression of leukemia [45], breast cancer [46], cervical cancer [47], lung cancer [48], and other cancers [49]. However, research on its therapeutic potential in non-oncological diseases remains limited. Huang et al. [50]. demonstrated that in a model of metabolic dysfunction-associated fatty liver disease, LDHB exacerbates hepatic steatosis by promoting the conversion of lactate to pyruvate, thereby providing substrates for de novo lipogenesis; conversely, inhibition of LDHB effectively alleviates hepatic lipid deposition. In kidney diseases, inhibiting *LDH* gene expression has been demonstrated to reduce LPS-induced damage in renal tubular epithelial cells. In this study, observations from in vivo AKI models, along with in vitro cellular injury experiments that model key pathogenic insults in renal cells, collectively confirmed that targeting LDHB reduces lactate production, alleviates cellular injury, and inhibits inflammatory cytokine expression. These findings suggest a promising therapeutic strategy for AKI. Admittedly, in this study our intervention primarily targets upstream glucose uptake via GLUT1 inhibition. However, alternative metabolic routes may bypass GLUT1-dependent glycolysis. These include the pentose phosphate pathway and the utilization of triose phosphates, both of which can influence redox balance independently of glucose uptake. Therefore, our data provide preliminary functional evidence for the involvement of lactate metabolism in AKI rather than strict mechanistic causality. Future studies should employ more specific genetic approaches, such as tubule-specific conditional knockout of *LDHB*, to definitively establish the causal role of *LDHB*.

## 5. Conclusions

In conclusion, through multi-omics analysis and multilevel experimental validation, this study highlights the importance of lactate metabolism, particularly involving LDHB, in AKI pathology. Our findings provide a novel perspective for understanding metabolic dysregulation in AKI and suggest that targeting LDHB to disrupt lactate metabolism dysregulation represents a promising therapeutic strategy for this condition, though preclinical evaluation of its safety and efficacy is required before clinical application.

## Data Availability

The datasets analyzed during the current study are available in the GEO database (https://www.ncbi.nlm.nih.gov/geo/ (accessed on 11 December 2025)), MSigDB database (https://www.gsea-msigdb.org/gsea/msigdb/index.jsp (accessed on 11 December 2025)), KEGG database (www.kegg.jp/kegg/kegg1.html (accessed on 11 December 2025)), GENEMANIA database(https://genemania.org/ (accessed on 11 December 2025)).

## Supplementary Materials

The following supporting information can be downloaded at: https://www.mdpi.com/article/10.3390/metabo16060434/s1, Figure S1: Quality control and clustering of single-cell data; Table S1: Summary of data sources and experimental details.

## Abbreviations

The following abbreviations are used in this manuscript:

AKI	Acute Kidney Injury
LMRGs	Lactate Metabolism-Related Genes
DC	Collecting Duct
bIRI	bilateral Ischemia/Reperfusion Injury
LPS	Lipopolysaccharide
H/R	Hypoxia/Reoxygenation
CKD	Chronic Kidney Disease
ICU	Intensive Care Unit
