# Supplementary material for: Lactate Metabolism Dysregulation Drives the Pathogenesis of Acute Kidney Injury
Source: Metabolites. 2026 Jun 22;16(6):434. doi: 10.3390/metabo16060434 (PMC13303532; doi:10.3390/metabo16060434)
Supplement: Supplementary file 1 [file metabolites-16-00434-s001.zip › Figure S1 Quality control and clustering of single-cell data.pdf]

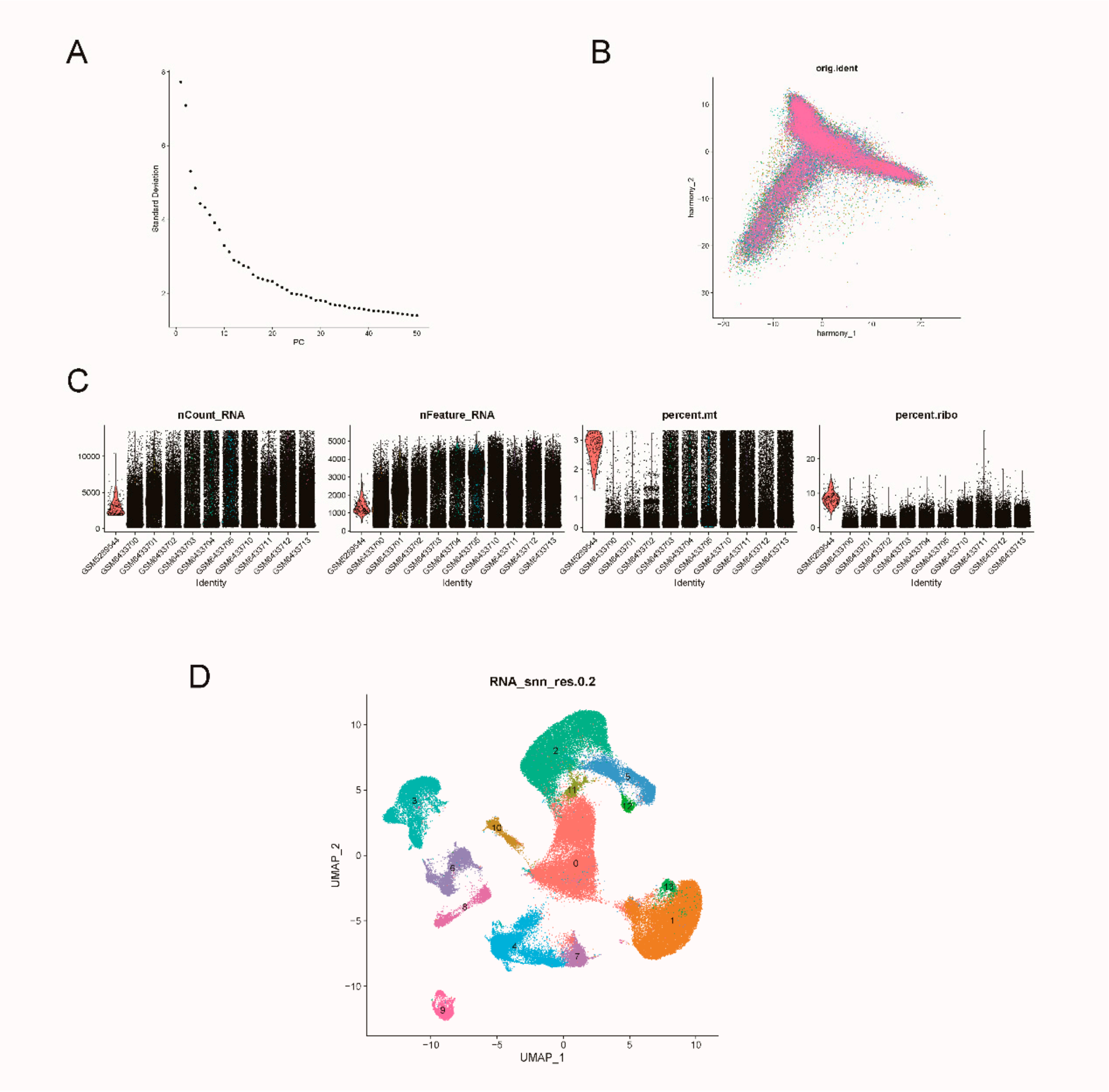

Figure S1. Quality control and clustering of single-cell data. (a) Elbow plot for determining the number of principal components. (b) Results after batch effect removal using the Harmony algorithm. (c) Violin plots showing the distribution of the number of genes (nFeature_RNA), number of transcripts (nCount_RNA), percentage of mitochondrial genes (percent.mt), and percentage of ribosomal genes (percent.ribo) per sample after quality control. (d) UMAP plot illustrating the cellular clusters.
